# Supplementary material for: Fossil black smoker yields oxygen isotopic composition of Neoproterozoic seawater
Source: Nat Commun. 2018 Apr 13;9:1453. doi: 10.1038/s41467-018-03890-w (PMC5899122; doi:10.1038/s41467-018-03890-w)
Supplement: Supplementary file 1 — Supplementary Information [file 41467_2018_3890_MOESM1_ESM.pdf]

Fossil black smoker yields oxygen isotopic composition of Neoproterozoic seawater

F. Hodel*[1,2], M. Macouin[1], R.I.F. Trindade[2], A. Triantafyllou[3], J. Ganne[1], V. Chavagnac[1], J. Berger[1], M. Rospabé[1], C. Destrigneville[1], J. Carlut[4], N. Ennih[5], P. Agrinier[4]

[1] Géosciences Environnement Toulouse (GET), Observatoire Midi Pyrénées, Université de Toulouse, CNRS, IRD, UPS, 31400 Toulouse, France

[2] Departamento de Geofísica, Instituto de Astronomia, Geofísica e Ciências Atmosféricas, Universidade de São Paulo, 05508-090 São Paulo, Brazil

[3] Laboratoire de Planétologie et Géodynamique, UMR-CNRS 6112, Université de Nantes, 44322 Nantes, France

[4] Institut de Physique du Globe de Paris, Université Sorbonne Paris Cité, Université Paris Diderot, CNRS, UMR 7154, 75005 Paris, France

[5] EGGPG, Département de Géologie, Faculté des Sciences, Université Chouaib Doukkali, 24000 El Jadida, Morocco

Correspondence to: F. Hodel, florent.hodel@hotmail.fr

**Fract. Law** (Zheng and Simon, 1991, Eur. J. Mineral.)[1]		
**$1000\ln\alpha mt\text{-}w = 3.02 \times 10^6 / T^2 - 12.00 \times 10^3 / T + 3.31$**		
**T (°C)**		**277**
**T (K)**		**550.15**
**Error On T**		**10**
**1000lnαmt-w**		**-8.52**
**Error on fractionation**		**0.36**
**Sample**	**$\delta^{18}O$ magnetite**	**$\delta^{18}O$ fluid**
**AA01**	-8.96	
**AA37F**	-8.16	
**AA37S**	-9.33	
**AA47**	-9.27	
**AA57**	-9.02	
**Mean**	**-8.95**	**-0.42**
**Std. Deviation (σ)**	0.47	
**2σ**	**0.93**	
**Std. Error**	0.21	
**2 Std. Error**	**0.42**	
**Error on fluid**		**0.55**
**Hydrothermal fluid to seawater shift (from compilation of 120 current black smokers)**		
**Mean $\Delta^{18}O_{BSfluid\text{-}PDseawater}$**	**2σ**	
**0.91**	**0.81**	
Jean-Baptsite et al. 1997. (Geochimica et Cosmochimica Acta)[2]		
Bach and Humphris, 1999. (Geology)[3]		
Reeves et al. 2011. (Geochimica et Cosmochimica Acta)[4]		
James et al. 2014. (Geochimica et Cosmochimica Acta)[5]		
**At 277 +/- 10 °C**		
**$\delta^{18}O$ NEOPROT. BOTTOM SEAWATER**		
**-1.33**	**(+/-)**	**0.98**

Supplementary Table 1: Isotopic data and calculations.

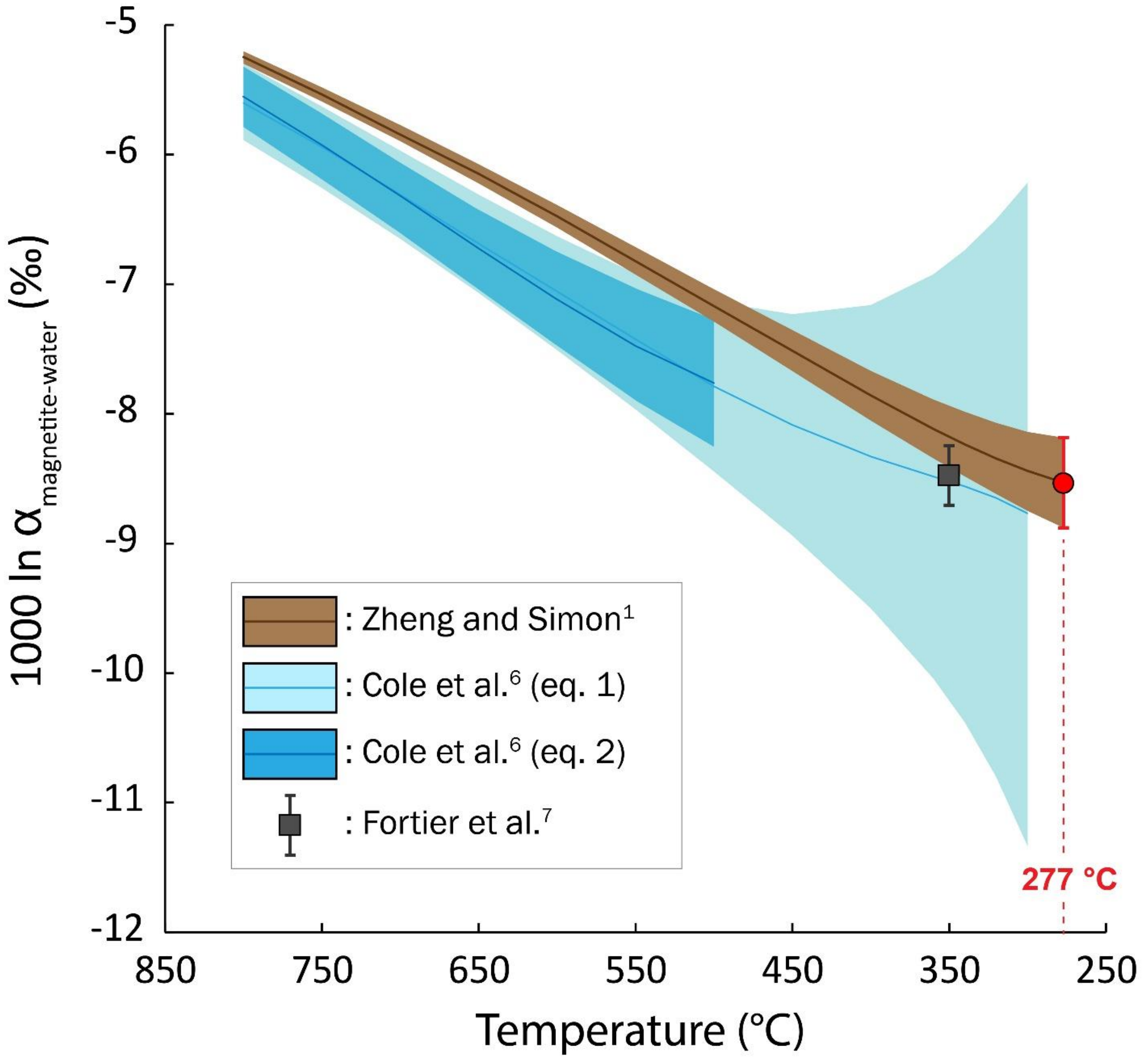

Supplementary Figure 1: Water-magnetite fractionation law selection. Graphical representation of water-magnetite fractionation factor as a function of temperature for the laws of Zheng and Simon[1] and Cole et al.[6] compared with the measured value of Fortier et al.[7]. Uncertainties were calculated for an uncertainty of 10 °C on the temperature. The uncertainty for the unique value at 350 ° C given by Fortier et al.[7] is specified in their study (±0.23‰).

Supplementary References

1. Zheng, Y.-F. & Simon, K. Oxygen isotope fractionation in hematite and magnetite : a theoretical calculation and application to geothermometry of metamorphic iron-formations. *Eur. J. Mineral.* **3**, 877–886 (1991).
2. Jean-Baptiste, P., Charlou, J. L. & Stievenard, M. Oxygen isotope study of mid-ocean ridge hydrothermal fluids: Implication for the oxygen-18 budget of the oceans. *Geochim. Cosmochim. Acta* **61**, 2669–2677 (1997).
3. Bach, W. & Humphris, S. E. Relationship between the Sr and O isotope compositions of hydrothermal fluids and the spreading and magma-supply rates at oceanic spreading centers. *Geology* **27**, 1067 (1999).
4. Reeves, E. P. et al. Geochemistry of hydrothermal fluids from the PACMANUS, Northeast Pual and Vienna Woods hydrothermal fields, Manus Basin, Papua New Guinea. *Geochim. Cosmochim. Acta* **75**, 1088–1123 (2011).
5. James, R. H. et al. Composition of hydrothermal fluids and mineralogy of associated chimney material on the East Scotia Ridge back-arc spreading centre. *Geochim. Cosmochim. Acta* **139**, 47–71 (2014).
6. Cole, D. R. et al. An experimental and theoretical determination of oxygen isotope fractionation in the system magnetite-H2O from 300 to 800°C. *Geochim. Cosmochim. Acta* **68**, 3569–3585 (2004).
7. Fortier, S. M. et al. Determination of the magnetite-water equilibrium oxygen isotope fractionation factor at 350°C: A comparison of ion microprobe and laser fluorination techniques. *Geochim. Cosmochim. Acta* **59**, 3871–3875 (1995).
