# Supplementary material for: Fossil black smoker yields oxygen isotopic composition of Neoproterozoic seawater
Source: Nat Commun. 2018 Apr 13;9:1453. doi: 10.1038/s41467-018-03890-w (PMC5899122; doi:10.1038/s41467-018-03890-w)
Supplement: Supplementary file 3 — Description of Additional Supplementary Files [file 41467_2018_3890_MOESM3_ESM.docx]

**Description of Additional Supplementary Files**

File Name: Supplementary Data 1

Description: Whole-rock major oxide and trace element concentrations in serpentinites from North Aït Ahmane, geochemical reference materials and blanks (Sheet 1). Compilation of whole-rock REE concentrations in serpentinites and variably serpentinized peridotites from abyssal (including black smoker related serpentinites) and supra-subduction environments reported in the literature.

File Name: Supplementary Data 2

Description: Mineral chemistry of chlorites and associated thermometry for the North Aït Ahmane serpentinites.
